# High Surface Phonon-Polariton in-Plane Thermal Conductance along Coupled Films

**DOI:** 10.3390/nano10071383

**Published:** 2020-07-15

**Authors:** Saeko Tachikawa, Jose Ordonez-Miranda, Yunhui Wu, Laurent Jalabert, Roman Anufriev, Sebastian Volz, Masahiro Nomura

**Affiliations:** 1 Institute of Industrial Science, The University of Tokyo, Tokyo 153-8505, Japan; yunhui@iis.u-tokyo.ac.jp (Y.W.); jalabert@iis.u-tokyo.ac.jp (L.J.); anufriev@iis.u-tokyo.ac.jp (R.A.); volz@iis.u-tokyo.ac.jp (S.V.); 2 Institut Pprime, CNRS, Universite de Poitiers, ISAE-ENSMA, F-86962 Futuroscope Chasseneuil, France; jose.ordonez@cnrs.pprime.fr; 3 Laboratory for Integrated Micro Mechatronic Systems/National Center for Scientific Research-Institute of Industrial Science (LIMMS/CNRS-IIS), The University of Tokyo, Tokyo 153-8505, Japan

**Keywords:** surface phonon-polaritons, multilayered structure, thermal conductance, polar materials

## Abstract

Surface phonon-polaritons (SPhPs) are evanescent electromagnetic waves that can propagate distances orders of magnitude longer than the typical mean free paths of phonons and electrons. Therefore, they are expected to be powerful heat carriers capable of significantly enhancing the in-plane thermal conductance of polar nanostructures. In this work, we show that a SiO${}_{2}$/Si (10 μm thick)/SiO${}_{2}$ layered structure efficiently enhances the SPhP heat transport, such that its in-plane thermal conductance is ten times higher than the corresponding one of a single SiO${}_{2}$ film, due to the coupling of SPhPs propagating along both of its polar SiO${}_{2}$ nanolayers. The obtained results thus show that the proposed three-layer structure can outperform the in-plane thermal performance of a single suspended film while improving significantly its mechanical stability.

## 1. Introduction

As the microfabrication technology has rapidly progressed and the integration density of electrical circuits has been enhanced dramatically, the local heating has become a critical issue because it can damage the electrical devices or lower their performance [1]. The nanoscale heat transfer has been investigated due to its importance in both fundamental physics and in semiconductor industries [2,3,4,5]. The thermal fluxes drop down with the sizes scaled down to nano-metric dimensions [6,7,8], therefore the thermal management is in great need.

Surface phonon-polaritons (SPhPs) attracted enormous attention in the past decade as an additional heat carrier. SPhPs are electromagnetic surface modes generated by the coupling between electromagnetic waves and optical phonons located at the interface of polar dielectric materials [9,10,11,12,13]. Studies on SPhPs have been conducted especially on polar materials, such as SiC [13,14,15,16], SiO${}_{2}$ [11,12,16,17,18,19], and hBN [20,21,22,23]. SPhPs propagate with infrared frequencies over distances in the range of hundreds of micrometers along the interface of nanostructures [18]. Therefore, they could be additional energy carriers capable of enhancing the in-plane thermal conductivity [17,19], especially when it decreases as the system size reduces to the nano scale [11,12,13]. Chen et al. have reported that the SPhP thermal conductivity of a 40 nm-thick thin film of amorphous SiO${}_{2}$ suspended in air is 4 Wm${}^{-1}$K${}^{-1}$ at 500 K, which is higher than its bulk phonon thermal conductivity [11]. An experimental demonstration was also conducted by Tranchant et al. [24]. They reported that the in-plane thermal conductivity of a SiO${}_{2}$ film increases by more than 50% of that of bulk SiO${}_{2}$ for a film thickness less than 50 nm.

However, a suspended thin film of a polar material is technically challenging to achieve and is not a practical geometry, thus a film on a substrate or a multilayered structure was then investigated [19,25,26]. Multilayered structures were found to achieve higher thermal conductivity than a single film for certain conditions [25]. Lim et al. [26] developed an analytical algorithm to scan the crucial parameters to optimize materials in multilayered configurations.

Here, we report SPhP dispersion relation of a structure consisting of a 10 μm-thick silicon (Si) layer sandwiched between two SiO${}_{2}$ layers (the three-layer structure). We demonstrate that this configuration exalts the coupling between polaritonic layers inside the Si film. When the Si layer thickness is within the cross-plane decay length of SPhPs in each polar film, the surface polaritons are indeed expected to couple without diminishing completely in the cross-plane direction and to carry thermal energy inside the system efficiently in the in-plane direction. Previous studies were focused on very thin nanofilms, where the SPhP dispersion relation was linearized and yielded only one solution (branch) [19,25,26]. In this study, we calculate the SPhP dispersion relation without using any analytical simplification and find several branches contributing to the SPhP thermal conductance. We found a higher thermal conductance due to SPhPs for the three-layer structure compared to that of a single SiO${}_{2}$ film thicker than 150 nm. This conductance becomes more than ten times higher for SiO${}_{2}$ films thicker than 600 nm. The novelty of this work lies in the discovery that the coupling of SPhPs propagating in each SiO${}_{2}$ layer of the SiO${}_{2}$/Si/SiO${}_{2}$ structure is able to enhance its ability to transport thermal energy, which results in a thermal conductance higher than the corresponding one of a single SiO${}_{2}$ film. This work provides insights into the design of the SPhPs supporting structures of large dimensions, which are mechanically stable, but can still yield a high SPhPs in-plane thermal conductance. Note that, throughout the manuscript, the contribution of acoustic phonons are not considered in the thermal conductance calculations.

## 2. Method and Model

**Figure 1:**
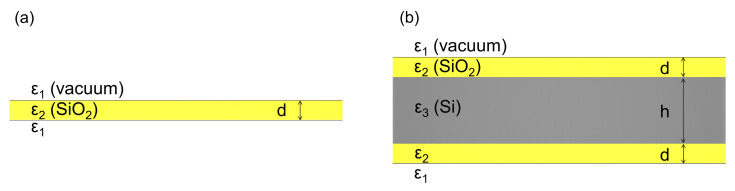
Scheme of (**a**) a single SiO${}_{2}$ film and (**b**) a three-layer structure (SiO${}_{2}$/Si/SiO${}_{2}$).

The SPhP dispersion relation of the three-layer structure, shown in Figure 1b, is derived in order to calculate the in-plane thermal conductance of the structure due to SPhPs.

Figure 1a shows the scheme of a single suspended film of thickness *d*, surrounded by vacuum, and Figure 1b displays the specific structure of a Si layer of thickness *h*, sandwiched between SiO${}_{2}$ films of thickness *d*. The SiO${}_{2}$ films and Si are assumed to be nonmagnetic (Relative magnetic permeability μ=1). The SiO${}_{2}$ has a frequency-dependent relative permittivity $\epsilon_{2}\left(\omega \right)$, while Si has a relative permittivity of $\epsilon_{3}=11.7$, which assumed as non-absorptive in the infrared range, surrounded by non-absorptive media with a permittivity of $\epsilon_{1}$ such as vacuum. The thermal conductance generated by SPhPs in this structure *G* is given by [11],

(1) $$G=\frac{W}{4\pi L}\int_{0}^{\infty }ћ\omega \Lambda \beta_{R}\frac{\partial f_{0}}{\partial T}d\omega .$$

where $f_{0}$ is the Bose-Einstein distribution function, *T* the temperature of the structure, and *ℏ* the reduced Planck’s constant, ω is the angular frequency, *W* and *L* are the system width and length, respectively. We assume the in-plane wave vector along the interfaces as a complex wave vector, denoted β as $\beta =\beta_{R}$+$i\beta_{I}$. The propagation length of SPhPs Λ is defined by

(2) $$\Lambda =\frac{1}{2\beta_{I}}.$$

By solving Maxwell’s equations with proper boundary conditions at each interface, we can obtain the dispersion relation for β of the three-layer structure as [25,27]

(3) $$\tanh\left(p_{3}h\right)=-\frac{2S_{23}\left[S_{12}+\tanh\left(p_{2}d\right)\right]\left[S_{12}\tanh\left(p_{2}d\right)+1\right]}{{\left[S_{12}+\tanh\left(p_{2}d\right)\right]}^{2}S_{23}^{2}+{\left[S_{12}\tanh\left(p_{2}d\right)+1\right]}^{2}}.$$

where $S_{nm}=p_{n}\epsilon_{m}/p_{m}\epsilon_{n}$, the transverse wave vector $p_{n}$ in the medium referred to as by *n* is

(4) $$p_{n}^{2}=\beta^{2}-\epsilon_{n}k_{0}^{2}.$$

with $\epsilon_{n}$ being the relative permittivity of medium *n* = 1, 2, 3, and the wave vector in vacuum $k_{0}=\omega /c$, where *c* is the speed of light in vacuum. This dispersion relation leads to the in-plane wave vector β. Note that we did not use any simplification when solving this dispersion relation, therefore the term $\tanh(p_{3}h)$ yields more than one solution. It means that several SPhPs branches exist in this structure. Knowing this β, one can obtain the in-plane thermal conductance of this structure by summing up the thermal conductances given by each branch.

**Figure 2:**
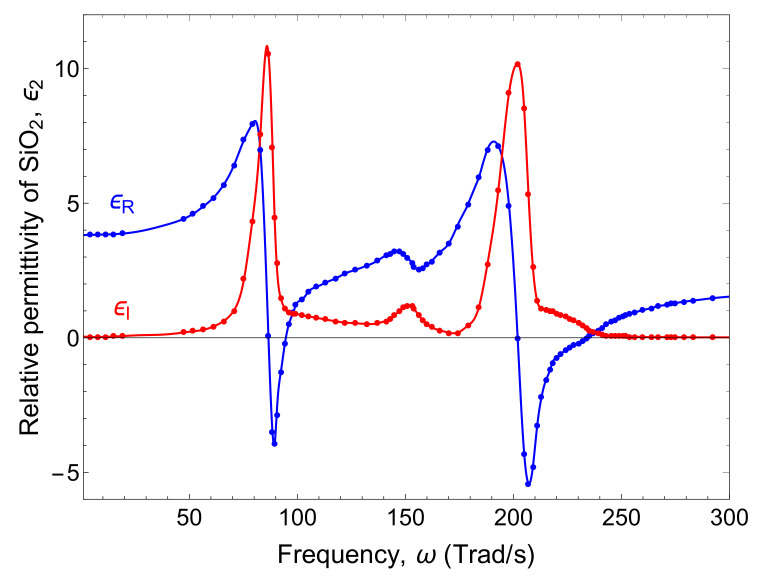
Real and imaginary parts of the relative permittivity of SiO${}_{2}$ reported in Ref [28].

Here, we consider silicon dioxide (SiO${}_{2}$) as the polariton active media. The relative permittivity of SiO${}_{2}$ is complex and frequency-dependent. The real part $\epsilon_{R}$ and imaginary part $\epsilon_{I}$ of the relative permittivity $\epsilon_{2}$ (ω) of SiO${}_{2}$ can be obtained from experimental data reported for the complex index of refraction [28], as shown in Figure 2.

The main peaks of $\epsilon_{I}$ at about 87 Trad/s and 202 Trad/s indicate that the SiO${}_{2}$ absorbs significant energy from the electromagnetic field at those frequencies. By contrast, the minimum of $\epsilon_{I}$ at 174 Trad/s shows that these fields can propagate relatively large distances within the SiO${}_{2}$, at this frequency. On the other hand, the two dips of $\epsilon_{R}$ at 89 Trad/s and 207 Trad/s are associated with the SPhP resonance frequencies and therefore the major contribution to the SPhP thermal conductance is expected to arise from the vicinity of the minimum of $\epsilon_{I}$ and the main dip of $\epsilon_{R}$, as shown below.

## 3. Results

**Figure 3:**
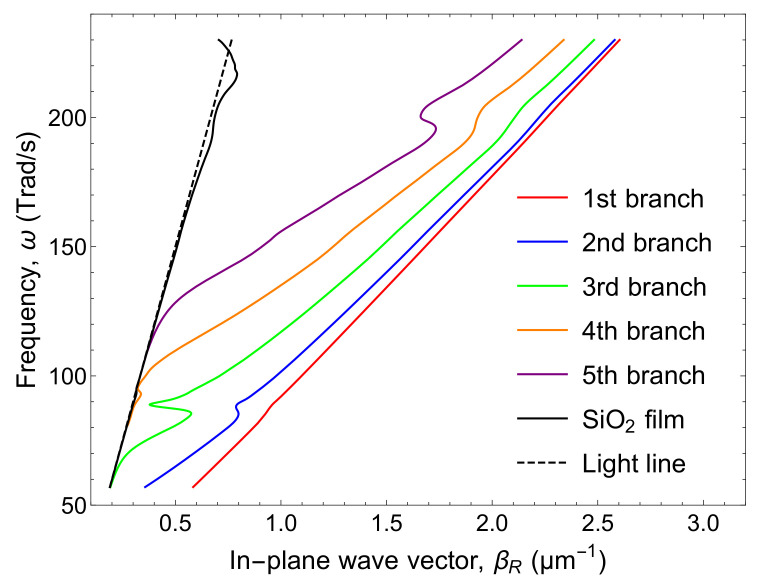
Dispersion relations of SPhPs propagating along a SiO${}_{2}$ (1 μm)/Si (10 μm)/SiO${}_{2}$ (1 μm) structure.

The dispersion relation of $\beta_{R}$ is plotted in Figure 3. There are five branches, named as the 1st–5th branches, within this frequency range. All the branches are below the light line, which means that they are evanescent in the cross-plane direction in this frequency range.

**Figure 4:**
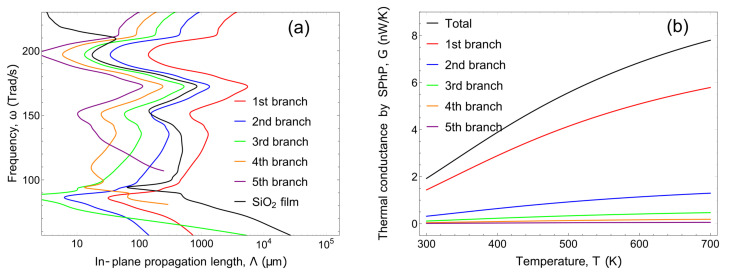
(**a**) The in-plane propagation length and (**b**) thermal conductance of SPhPs. The structure consists of the layered system, SiO${}_{2}$ (1 μm)/Si (10 μm)/SiO${}_{2}$ (1 μm).

The in-plane propagation length of each branch is plotted in Figure 4a. The first branch has the longest propagation length at frequencies above 90 Trad/s and this latter length is even longer than the in-plane propagation length of SPhPs in a single SiO${}_{2}$ film. This behavior leads to the high thermal conductance of the first branch in the structure.

The thermal conductance of each branch is plotted in Figure 4b. The first branch with the longest propagation length shows the largest thermal conductance. However, the thermal conductance given by the second branch is equivalent to approximately 16% of the total thermal conductance, and the contribution of the second branch is not negligible.

**Figure 5:**
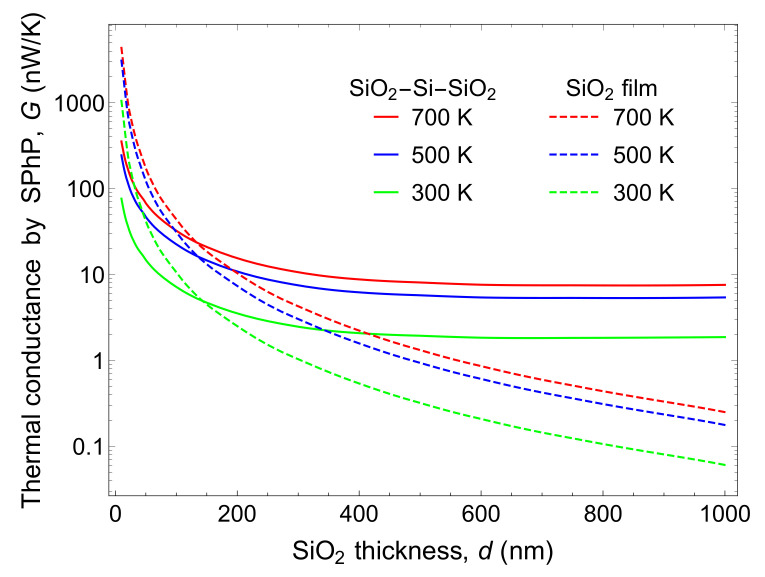
A comparison of the SPhPs thermal conductances of the SiO${}_{2}$/Si (10 μm)/SiO${}_{2}$ three-layer structure and of a single SiO${}_{2}$ film at 300, 500 and 700 K, for different SiO${}_{2}$ thicknesses.

For comparison, we calculated the thermal conductance dependence on the SiO${}_{2}$ thickness, shown in Figure 5. The SiO${}_{2}$ thickness is varied from 1 μm down to 10 nm and both the thermal conductance by SPhPs of the three-layer structure and of the single SiO${}_{2}$ film are plotted. The thermal conductance of the three-layer structure is higher than that of the single SiO${}_{2}$ film for SiO${}_{2}$ film thickness > 150 nm. When the SiO${}_{2}$ film is thicker than 600 nm, the thermal conductance is more than one order of magnitude higher than that of the single SiO${}_{2}$ film.

## 4. Discussion

For most of the studies of SPhPs, thin enough layers ($|p_{3}|h\ll 1$, $|p_{2}|d\ll 1$) were considered. This justified the assumption of tanh(x)→x, reducing the dispersion relation equation into a linear relation yielding a single solution. In this study, Si thickness of 10 μm is considered as not thin enough to apply the assumption, so that the equation was calculated numerically, resulting in several different solutions or branches. The higher the order of the branch, the shorter the propagation length. Therefore, each branch contributes to thermal transport at different levels. The first branch has the longest propagation length over nearly all the frequency range, even longer than that of a 1 μm-thick single film. It provides the most significant contribution of approximately 70% to the thermal conductance. The second branch and the third branch contribute to ∼16% and ∼5% of the thermal conductance, respectively. In the structure of relatively large dimensions, there is more than one branch to take into account in the calculation of the thermal conductance. The branches with longer propagation lengths pushed the thermal conductance higher than that of the single SiO${}_{2}$ film case. However, the reduction of SiO${}_{2}$ thickness enhances the thermal transport by SPhP in in-plane due to less absorption in the polar materials and this effect has less impact on the three-layer structure, for its low effective volume of the polar material among the total volume. Therefore, as plotted in Figure 5, the thermal conductance of the three-layer structure cannot defeat that of the single SiO${}_{2}$ film, for SiO${}_{2}$ thickness smaller than 150 nm. Yet, this result shows that bulk structure can give higher thermal conductance by carefully choosing the material and the configuration without thinning the suspended film to create a mechanically unstable and non-realistic structure. As for Si thickness dependence, as the thickness of the Si layer reduces, the coupling between the SPhPs propagating along the top and bottom SiO${}_{2}$ layers strengthens, with both layers tend to act like a single thicker one, which increases the absorption of energy by SiO${}_{2}$ and hence decreases the SPhP thermal conductance.

**Figure 6:**
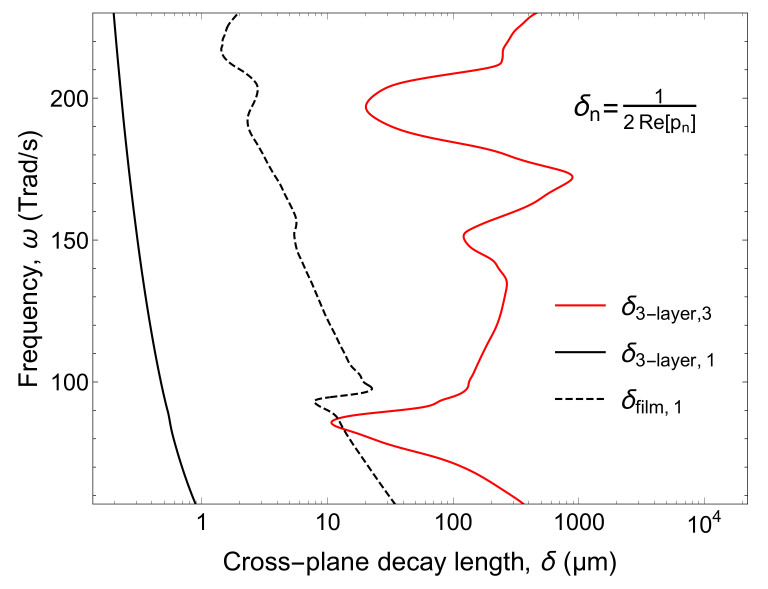
Cross-plane decay length in different media: Si (red solid line) and vacuum (black solid line) of the three-layer structure, and vacuum in the vicinity of the single film structure (black dashed line).

**Figure 7:**
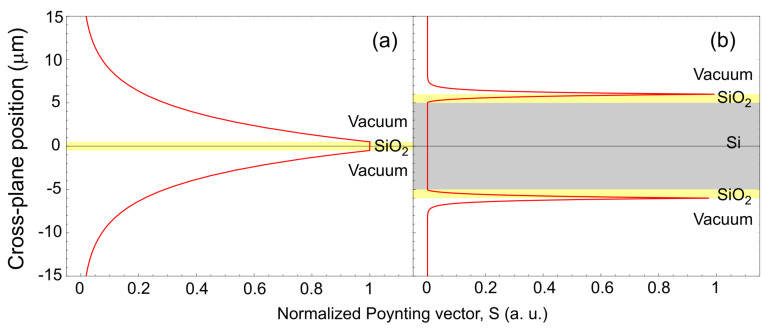
Normalized Poynting vector along the cross-plane direction for (**a**) the single SiO${}_{2}$ film and (**b**) the three-layer structure surrounded by vacuum.

We also calculated the cross-plane wave vectors in each media of the three-layer structure and compared them to the one of the single SiO${}_{2}$ film case for SiO${}_{2}$ thickness of 1 μm. In Figure 6, the cross-plane decay length δ in Si ($\delta_{3-layer,3}$) and in vacuum at the vicinity of the three-layer structure ($\delta_{3-layer,1}$) are plotted in addition to the δ in vacuum at the vicinity of the single SiO${}_{2}$ film ($\delta_{film,1}$). It shows that $\delta_{3-layer,3}$ is longer than $\delta_{film,1}$, indicating that SPhPs in the top SiO${}_{2}$ layer and the SPhPs at the bottom SiO${}_{2}$ layer are coupled inside Si. Note that $\delta_{3-layer,3}$ is very long so that almost not decaying within 10 μm thick Si. On the other hand, $\delta_{3-layer,1}$ is shorter compared to $\delta_{film,1}$. Figure 7 shows the Poynting vector distribution along the cross-plane direction at ω= 174 Trad/s. As predicted in Figure 6, for the three-layer structure, the wave in the cross-plane direction decays more quickly than for the single SiO${}_{2}$ film case and is more confined at the interface between SiO${}_{2}$ and vacuum. The energy is more distributed inside the structure instead of spreading into the vacuum surroundings. When the in-plane thermal conductance by SPhPs of the structure is concerned, SPhPs of two films coupled inside Si which propagates without being absorbed in Si, and energy strongly confined around the structure can effectively yield higher value than the summation of the SPhPs thermal conductance of two individual single SiO${}_{2}$ films.

The three-layer structure supports SPhPs with long propagation lengths, and thus yields a significant SPhPs thermal conductance even with relatively thick SiO${}_{2}$ layers which are normally not effective in enhancing SPhPs thermal transport. Although the SiO${}_{2}$ thickness reduction leads to a substantial impact in the single film case and the thermal conductance for the single film exceeds that of the three-layer structure with SiO${}_{2}$ thinner than 150 nm, suspended films with this range of thicknesses are difficult to handle and barely realistic in terms of applications.

## 5. Conclusions

We have calculated the dispersion relation of SPhPs in a SiO${}_{2}$/Si (10 μm)/SiO${}_{2}$ three-layer structure involving two coupled polaritonic nanofilms. Several branches exist in the structure with different in-plane propagation lengths and the total thermal conductance is a sum of the thermal conductance given by each branch. The three-layer structure exhibits higher SPhP thermal conductance than a single SiO${}_{2}$ film when SiO${}_{2}$ is thicker than 150 nm in both structures. Furthermore, the SPhP thermal conductance of the structure is ten times higher than the one of a single SiO${}_{2}$ film thicker than 600 nm. Finally, we have demonstrated that the energy decay in the cross-plane direction inside Si was almost negligible, whereas more confinement in the vicinity of the SiO${}_{2}$ layer for the structure has been found through a cross-plane wave vectors analysis. This work shows that the coupling of two polaritonic nanofilms generates a substantial gain in electromagnetic heat flux, while ensuring strong mechanical stability.
